# Psychometric Properties of the Family Inventory of Resources for Management in a Sample of Iranian Family Caregivers of Cancer Patients

**DOI:** 10.1155/2016/1401645

**Published:** 2016-12-29

**Authors:** Seyed Reza Mirsoleymani, Camelia Rohani, Mahsa Matbouei, Malihe Nasiri, Parvaneh Vasli

**Affiliations:** ^1^School of Nursing and Midwifery, Shahid Beheshti University of Medical Sciences, Valiasr st., Niayesh Crossroad, Tehran, Iran; ^2^Department of Nursing, School of Nursing and Midwifery, Shahid Beheshti University of Medical Sciences, Valiasr st., Niayesh Crossroad, Tehran, Iran

## Abstract

*Objective.* The aim of this study was to investigate the psychometric properties of the Family Inventory of Resources for Management (FIRM) in a sample of family caregivers of cancer patients.* Methods.* In this methodological study, construct validity of the FIRM was evaluated by known groups and convergent validity in a convenience sample of family caregivers of cancer patients (*n* = 104) referred to the outpatient oncology wards of five educational hospitals in Tehran from January to April 2016. Reliability was determined by assessing the internal consistency and stability of the instrument.* Results.* The known-groups findings showed that there is a significant difference between the scores of the FIRM in family caregivers with different levels of caregiver burden (*p* < 0.001). Also, the results of convergent validity showed that there is a moderate negative correlation (*r* = −0.50; *p* < 0.001) between the total scores of the FIRM and the scores of the caregiver burden inventory (CBI). The FIRM showed a good internal consistency (*α* = 0.85) and a good stability of the test-retest reliability result.* Conclusions.* There is a sound psychometric basis for the use of the Persian translation of the FIRM for family studies in the Iranian population.

## 1. Introduction

A family crisis is an interruption in the routine of a family that leads to disorganization of the family homeostasis. When a crisis occurs in a family, the family resources and coping strategies to manage stress may not be effective. Resources are those features and supports that are accessible for use by the family in crisis situations [1]. Family resources as the main protective factor in a crisis situation [2] are the key construct of the Resiliency Model of Family Stress, Adjustment, and Adaptation [3]. This model shows that, in unpredictable events in the family system, when family resources are insufficient in handling the demands and recovering of family adaptation, crisis occurs. During a crisis, the family system should plan for recovering of the situation by making basic changes in the established patterns of functioning and management of the internal and external resources [4]. According to the Resiliency Model, cancer diagnosis and care management of the patient within the family create a situational crisis in the family system [5, 6]. As a consequence, the management of the family resources and the process of family adaptation are required throughout life's challenges [7].

Individual (intelligence, knowledge, proficiency, and personality features, such as optimism), family (decision-making skills, organizing, and problem-solving abilities), and social resources are accessible, and include personal supports (relatives and friends) and institutional supports [1]. In an effort to assess the family's collection of resources, the Family Inventory of Resources for Management (FIRM) was developed by McCubbin and colleagues. It is hypothesized that families with a larger collection of resources will manage more successfully and will be able to adapt better to stressful conditions. This instrument measures weaknesses and strengths of the family to access resources and identifies the resources that a family needs for empowerment [8]. Since no Persian instrument exists for measuring family resources, the present study was conducted. The aim of this study was to investigate the psychometric properties of the FIRM in a sample of Iranian family caregivers of cancer patients, following the translation process of the instrument.

## 2. Methods

### 2.1. Design

This is a methodological study with a cross-sectional design which was conducted in two phases. In phase 1, translation and determination of the face and content validity of the FIRM were done. In phase 2, the psychometric properties of the FIRM, containing the known-groups technique, convergent validity, and reliability were examined.

### 2.2. Instruments

#### 2.2.1. Family Inventory of Resources for Management (FIRM)

The FIRM, as a self-report instrument was designed to measure available family resources to assist family adaptation to stressful life events in the areas of personal, family system, and internal resources as well as social support. It is a 69-item instrument comprising four subscales: esteem and communication (family strengths I, 15 items), mastery and health (family strengths II, 20 items), extended family social support (4 items), and financial well-being (16 items). Items that provide information about the resources of financial support and social desirability are not considered as a main part of the instrument. All responses for items of the subscales, ranging from 0 (not at all) to 3 (very well), were summed for a total subscale score. The total scores of the four subscales provide a total FIRM score, with a higher score showing generally greater resources. The English version of the FIRM demonstrated acceptable validity and reliability (*α* = 0.89) [8].

#### 2.2.2. Caregiver Burden Inventory (CBI)

The CBI, as a self-report instrument, measures caregiver burden among caregivers of patients with chronic diseases. This instrument consists of 24 items that are scored on a Likert scale from 0 (never) to 4 (nearly always). The CBI has five subscales: developmental burden (5 items), time dependence burden (5 items), physical burden (4 items), social burden (5 items), and emotional burden (5 items). The total score of the CBI ranges from 0 to 96, with higher scores indicating greater caregiver burden. Scores above 36 indicate a risk of burnout; scores close to or slightly above 24 indicate a need to seek some form of respite care [9]. A literature review indicates that the internal consistency of this instrument is between 0.92 and 0.94 [10]. Cronbach's alpha coefficient of the Persian version of the CBI is 0.90 [11].

#### 2.2.3. Demographic-Clinical Information Questionnaire

Demographic and clinical data were obtained by a questionnaire with six questions, containing family caregivers' age, gender, and education as well as patients' age, gender, and type of cancer during a short interview and by the medical records.

### 2.3. Phase 1: Translation, Face, and Content Validity

Permission for translation was first obtained from the instrument developer, Dr. Hamilton McCubbin. The FIRM was then translated, in accordance with standard guidelines [12, 13]. The English version of the FIRM was translated into the Persian language by two bilingual Iranians with nursing expertise. Two other Iranians with the same educational level then performed blind back-translations. All versions of the FIRM were reviewed by the research team and compared with the original version.

Face and qualitative content validity of the Persian version of the FIRM were evaluated by an expert panel composed of 10 faculty members of the School of Nursing and Midwifery at Shahid Beheshti University of Medical Sciences. They were specialists in nursing and experts in research methodology. In accordance with the suggestions of the expert panel, the scale content validity index (S-CVI) was calculated. A score of greater than 0.80 indicates good validity of the instrument [14]. Subsequently, face validity of the FIRM was assessed by a convenience sample of family caregivers of cancer patients (*n* = 10), who were asked to evaluate the items of the instruments and judge them for readability and clarity.

### 2.4. Phase 2: Psychometric Tests

#### 2.4.1. Known-Groups and Convergent Validity

For hypothesis testing in evaluation of construct validity of the FIRM, the known-groups and convergent validity were estimated. The known-groups technique was based on a hypothesis and supposed to measure the ability to discriminate between two groups who are known or expected to differ with regard to the construct of interest. Convergent validity measures the degree to which a measurement correlates with measurement scores of a convergent construct, when there is no gold standard [14]. For known-groups validity, a hypothesis was released; it was that the family caregivers with having access to fewer resources for managing a crisis experience a greater burden than the other caregivers, based on prior studies [8]. The samples were divided into two groups, according to the level of caregiver burden, and the scores of the FIRM were compared between the groups by independent *t*-test. The convergent validity of the FIRM was evaluated by testing the correlation between the FIRM and the CBI scores using the Pearson correlation coefficient. In accordance with the existing evidence, a main hypothesis was formulated: there is a slight to moderate negative correlation between the scores of the FIRM and the CBI.

In this study the sample size was calculated to be 100 participants based on related studies [4] and by the statistical formula (*α* = 0.05, *d* = 2); due to attrition risk, 120 participants were calculated. By convenience sampling from January to April 2016, 120 family caregivers of cancer patients referred to chemotherapy to the outpatient oncology wards of five educational hospitals affiliated to Shahid Beheshti University of Medical Sciences in Tehran were studied. Of these, 16 caregivers declined to complete the instruments and were excluded from the study (response rate: 86.7%).

#### 2.4.2. Reliability

The reliability of the FIRM was determined by the internal consistency and stability of the instrument. Thirty family caregivers of cancer patients separate from the main study sample completed the instrument and then repeated this two weeks later (*n* = 30). The reliability of the instrument was then determined by Cronbach's alpha coefficient and intraclass correlation coefficient (ICC). Cronbach's alpha coefficient was also calculated in the main sample of the study (*n* = 104).

### 2.5. Ethical Considerations

This study was approved by the ethics committee of the School of Nursing and Midwifery at Shahid Beheshti University of Medical Sciences (Code: IR.SBMU.PHNM.1394.209). All the participants were informed about the purpose of the study and their right to consent or refuse to participate and to withdraw from the study at any time without any consequences. All participants signed informed consent forms.

### 2.6. Data Analysis

The normal distribution of the main study variables was confirmed by the Kolmogorov-Smirnov test. The parametric analyses were chosen and all statistical analyses were conducted using SPSS version 21. The Pearson correlation coefficient was applied. A correlation below 0.20 is considered low, between 0.20 and 0.35 slight, 0.36–0.65 moderate, 0.66–0.85 high, and 0.86 and above considered very high [15]. A significant level was considered at *p* < 0.05.

## 3. Results

### 3.1. Descriptive Data

The mean ages of family caregivers and their patients were 40.3 ± 13.5 and 51.7 ± 15.3 years, respectively. Most of the family caregivers (55.8%) and their patients (68.3%) were women and 61.5% of the patients were diagnosed with breast cancer.  Table 1 shows the demographic information of family caregivers and their patients.

### 3.2. Results of Phase  1

The two Persian versions of the FIRM were compared with the original version and the differences were determined, so that the best translation could be selected. The face and content validity of the instrument were determined by the expert panel. The comments and suggestions of all expert panel members were considered and necessary changes were applied (items 5, 13, 19, 21, 46, 47, 55, 61, and 65 were changed). The scale content validity index (S-CVI) was 97% for the Persian version of the FIRM, 0.98 for the esteem and communication, 0.95 for the mastery and health, 0.96 for the extended family social support, and 0.99 for the financial well-being. Ten family caregivers of cancer patients (*n* = 10; 7 females and 3 males; mean age of 58.45 ± 6.8 years) evaluated satisfactory of the face validity of the Persian version of the FIRM.

### 3.3. Results of Phase  2

Significant differences in the total FIRM scores were determined between two groups of family caregivers with severe (CBI > 36) and no severe caregiver burden (CBI ≤ 36) using an independent *t*-test and the hypothesis was confirmed. The mean score of the FIRM for family caregivers who experienced greater caregiver burdens was significantly lower than for those of the other family caregivers. Therefore, the validity of the FIRM was supported by the known-groups technique. These families were determined to have access to poor resources for caring for a cancer patient in the family (Table 2).

The mean score of the FIRM and the CBI for family caregivers of cancer patients in this study was 112.1 ± 28.2 (range: 0–207) and 36.9 ± 19 (range: 0–96), respectively. To evaluate the convergent validity, the results of correlation between the scores of CBI and FIRM showed that there is a significant negative correlation between them (*r* = −0.50, *p* < 0.001) and our hypothesis for convergent validity was confirmed. Of the FIRM subscales, the mastery and health showed the strongest negative correlation with the total CBI score (*r* = −0.44, *p* = 0.01) (Table 3).

The results of reliability analyses supported a good internal consistency with the Cronbach's alpha coefficients of 0.91 and 0.85, respectively, in the small sample of the study (*n* = 30) and with the main study sample (*n* = 104) (Table 4). The ICC for the total score of the FIRM (ICC = 0.89) and its subscales (range 0.77–0.92) was shown in Table 4.

## 4. Discussion

The present study investigated the psychometric properties of the FIRM in a sample of Iranian family caregivers of cancer patients, following translation of the instrument. The Persian version of the FIRM appeared to be a valid and reliable instrument.

The face and content validity of the FIRM were approved. Satisfactory results were reported for the S-CVI of the instrument and all subscales by the expert panel. It is noticeable that in our study the family caregivers, who had access to fewer resources, experienced more burden, and the hypothesis of the known-groups validity was confirmed. This result is consistent with those of McCubbin et al. [8], in which the mothers of chronically ill children with fewer resources reported more problems with caregiving.

The results of this study showed that there was a moderate negative correlation between CBI and FIRM scores (*r* = −0.50, *p* < 0.001). Among the subscales of the FIRM, the mastery and health showed the strongest negative correlation with caregiver burden total scores (*r* = −0.44, *p* < 0.01). Thus, our hypothesis for convergent validity of the FIRM was confirmed. Some studies showed that the families with more resources had a better chance for managing the stressors and improving their family coherence [16]. Khamis [17] found that, in Palestinian families who are living in conflict areas, the mastery and health were significant predictors of psychological distress (*β* = −0.30, *p* < 0.001) and neuroticism (*β* = −0.25, *p* < 0.01) [16]. The “mastery” refers to the extent to which people can control their lives. A sense of mastery can protect people against stressful events directly and indirectly. For example, families with a stronger sense of mastery believe that they can handle all problems and control all unpredictable situations. Improvement in the health is also critical for providing care for a patient as a family member [18].

Overall, our results provided support for the reliability of the Persian version of the FIRM in the Iranian culture, both regarding internal consistency and stability. The FIRM, with Cronbach's alpha coefficient of 0.85, showed a good internal consistency. Among its subscales, the esteem and communication indicated the highest internal consistency with Cronbach's alpha coefficient of 0.85, and the extended family social support with Cronbach's alpha coefficient of 0.74 showed the least. Other studies reported similar results. Corwin et al. [19] conducted a study on US families with attention deficit hyperactivity disorder (ADHD) children. They reported Cronbach's alpha coefficient of the FIRM in mothers 0.95 and in fathers 0.87. Agonis [20] reported this value for families of patients with brain injury in Florida (*α* = 0.88). Developer of the FIRM, McCubbin et al. [8] reported Cronbach's alpha coefficient of 0.89 for this instrument and for all subscales more than 0.85 (except for the extended family social support with 0.62). Van Riper showed that all subscales of the FIRM (except for the extended family, social support with 0.65) had Cronbach's alpha coefficient score of more than 0.80 [21]. The test-retest results indicated that the FIRM was a well-established instrument during the time, parallel with other studies [4]. It can be concluded that despite the lack of studies in this area, it seems that the FIRM has a good theoretical base for family studies.

Regardless of the adequate sample size from different educational hospitals, the sample was a nonrandom selection of family caregivers with cancer patients. Thus, it was to some extent not a representative sample of the Iranian family caregivers of cancer patients. This may limit the external validity of the results and should be used cautiously. Also, it is important to note that this study does not complete the process of psychometrics but indicates that there is a sound psychometric basis for using the Persian version of the FIRM in family studies. In summary, the results of this study show that the Persian version of the FIRM is a valid and reliable instrument as demonstrated in a sample of Iranian family caregivers with cancer patients. More research is needed to establish advanced psychometric tests, such as factor analysis in different populations.

## 5. Conclusions

The results of this study showed that the validity and reliability of the Iranian version of the Family Inventory of Resources for Management (FIRM) are supported. It is a psychometrically sound instrument which is applicable to family studies with Iranian population.

## Figures and Tables

**Table 1:** Demographic and clinical variables in family caregivers and cancer patients (*n* = 104).

Variables	*n*	%
*Family caregivers' age*		
≤24	14	13.5
25–44	52	50.0
45–64	34	32.7
≥65	4	3.9
*Family caregivers' gender*		
Male	46	44.2
Female	58	55.8
*Family caregivers' education*		
Illiterate	4	3.8
Primary school	8	7.7
Secondary school	15	14.4
High school	49	47.1
University	28	26.9
*Patients' age*		
≤24	4	3.8
25–44	32	30.8
45–64	49	47.1
≥65	19	18.3
*Patients' gender*		
Male	33	45.3
Female	71	33.0
*Type of cancer *		
Breast	64	61.5
Prostate	5	4.8
Lung	9	8.7
Colorectal	17	16.3
Others	9	8.7

**Table 2:** Comparison of the FIRM mean scores, according to the level of caregiver burden in two groups of family caregivers of cancer patients (*n* = 104).

Variables	*n*	Mean score^*∗*^	SE
Caregivers with severe burden (>36)	50	101.10	25.53
Caregivers with no severe burden (≤36)	54	122.38	26.84

^*∗*^ *p* < 0.001, *t* = 4.13.

**Table 3:** Correlation between the score of the FIRM and the caregiver burden inventory (CBI) in family caregivers of cancer patients (*n* = 104).

	Family strengths I, esteem and communication	Family strengths II, mastery and health	Extended family social support	Financial well-being	FIRM total score
CBI	−0.38	−0.44	−0.30	−0.33	−0.50

Note:  all correlations are significant (*p* < 0.01).

**Table 4:** Cronbach's alpha coefficient and test-retest reliability of the FIRM.

FIRM	*α*(*n* = 104)	ICC^*∗*^(*n* = 30)
(1) Family strengths I, esteem and communication	0.85	0.82
(2) Family strengths II, mastery and health	0.88	0.80
(3) Extended family social support	0.74	0.77
(4) Financial well-being	0.75	0.92
(5) Total score	0.85	0.89

^*∗*^Intraclass correlation coefficient.
